# Association Between Exposure to Hurricane Irma and Mortality and Hospitalization in Florida Nursing Home Residents

**DOI:** 10.1001/jamanetworkopen.2020.19460

**Published:** 2020-10-06

**Authors:** David M. Dosa, Julianne Skarha, Lindsay J. Peterson, Dylan J. Jester, Nazmus Sakib, Jessica Ogarek, Kali S. Thomas, Ross Andel, Kathryn Hyer

**Affiliations:** 1 School of Public Health, Brown University, Providence, Rhode Island; 2 Warren Alpert Medical School, Brown University, Providence, Rhode Island; 3 Providence VAMC, Center of Innovation for Long Term Services and Supports, Providence, Rhode Island; 4 University of South Florida, School of Aging Studies, Tampa; 5 University of South Florida, Department of Industrial and Management Systems Engineering, Tampa

## Abstract

**Importance:**

Nursing home residents are at heightened risk for morbidity and mortality following an exposure to a disaster such as a hurricane or the COVID19 pandemic. Previous research has shown that nursing home resident mortality related to disasters is frequently underreported. There is a need to better understand the consequences of disasters on nursing home residents and to differentiate vulnerability based on patient characteristics.

**Objective:**

To evaluate mortality and morbidity associated with exposure to Hurricane Irma, a Category 4 storm that made landfall on September 10, 2017, in Cudjoe Key, Florida, among short-stay (<90-day residence) and long-stay (≥90-day residence) residents of nursing homes.

**Design, Setting, and Participants:**

Cohort study of Florida nursing home residents comparing residents exposed to Hurricane Irma in September 2017 to a control group of residents residing at the same nursing homes over the same time period in calendar year 2015. Data were analyzed from August 28, 2019, to July 22, 2020.

**Exposure:**

Residents who experienced Hurricane Irma were considered exposed; those who did not were considered unexposed.

**Main Outcome and Measures:**

Outcome variables included 30-day and 90-day mortality and first hospitalizations after the storm in both the short term and the long term.

**Results:**

A total of 61 564 residents who were present in 640 Florida nursing home facilities on September 7, 2017, were identified. A comparison cohort of 61 813 residents was evaluated in 2015. Both cohorts were mostly female (2015, 68%; 2017, 67%), mostly White (2015, 79%; 2017, 78%), and approximately 40% of the residents in each group were over the age of 85 years. Compared with the control group in 2015, an additional 262 more nursing home deaths were identified at 30 days and 433 more deaths at 90 days. The odds of a first hospitalization for those exposed (vs nonexposed) were 1.09 (95% CI, 1.05-1.13) within the first 30 days after the storm and 1.05 (95% CI, 1.02-1.08) at 90 days; the odds of mortality were 1.12 (95% CI, 1.05-1.18) at 30 days and 1.07 (95% CI, 1.03-1.11) at 90 days. Among long-stay residents, the odds of mortality for those exposed to Hurricane Irma were 1.18 (95% CI, 1.08-1.29) times those unexposed and the odds of hospitalization were 1.11 (95% CI, 1.04-1.18) times those unexposed in the post 30-day period.

**Conclusions and Relevance:**

The findings of this study suggest that nursing home residents are at considerable risk to the consequences of disasters. These risks may be underreported by state and federal agencies. Long-stay residents, those who have resided in a nursing home for 90 days or more, may be most vulnerable to the consequences of hurricane disasters.

## Key Points

**Question:**

Was exposure to Hurricane Irma associated with an increased risk of hospitalization and mortality among nursing home residents in the 30 and 90 days after the storm compared with a control group?

**Findings:**

In this cohort study of 61 564 nursing home residents exposed to Hurricane Irma and a control group of 61 813 nonexposed residents, the odds of a first hospitalization and mortality increased significantly at 30 and 90 days for those exposed. A long nursing home stay was associated with a greater risk for mortality compared with a short stay.

**Meaning:**

Findings suggest that prioritizing heightened emergency preparedness in disaster situations for nursing home residents is warranted.

## Teaser

This cohort study assess the association between exposure to Hurricane Irma and 30- and 90-day mortality and morbidity among short- and long-term residents of nursing homes in Florida.

## Introduction

Nursing home preparedness in the face of all hazard emergencies is a vital area of importance, particularly given the demonstrated mortality within long-term care facilities associated with the coronavirus disease 2019 (COVID-19) pandemic. Recent data show that nearly one-quarter of the US deaths related to COVID-19 have occurred in the long-term care setting.^1^ Previous research involving nursing home residents exposed to hurricane disasters demonstrates how vulnerable they are to disaster situations.^2,3^ Residents exposed to 1 of 4 Gulf of Mexico hurricanes (Katrina, Rita, Gustav, and Ike) showed significant increases in morbidity and mortality compared with nondisaster years. Furthermore, the number of deaths in the nursing home setting significantly exceeded those reported by health care officials using death certificate records.^2,3^

Despite this data spotlighting the vulnerability of nursing home residents to disasters, little is known about which residents are more susceptible to harm associated with exposure to a hurricane or other disaster. Previous research in community-living older adults has found that hurricane exposure is associated with increased emergency department visits and hospitalization for those with diabetes or those receiving dialysis.^4,5^ Similar research in the nursing home setting is limited. Studies of residents exposed to Hurricane Gustav found that posthurricane hospitalizations increased for the most functionally impaired residents and that death increased for those with dementia.^6,7^

There is a need to better understand the factors that increase residents’ vulnerability to morbidity and mortality in a disaster. Nursing home residents overall have a high level of morbidity. Nearly 50% have been diagnosed with Alzheimer disease or other dementias, 32% have diabetes, and nearly 40% have heart disease. However, most nursing homes serve 2 different populations, short-stay and long-stay residents. Residents admitted to a nursing home after a hospital stay for Medicare-covered skilled care or rehabilitation (up to a maximum of 89 days) are classified as short-stay. Short-stay residents may be discharged at the end of their stay, or they may remain at the nursing home as a long-stay resident. Long-stay residents, most covered by Medicaid, make up two-thirds of the nursing home population. Previous research has shown that long-stay residents are generally frailer than short-stay residents.^8,9^They are more likely than short-stay residents to have osteoporosis and to have been diagnosed with Alzheimer disease or other dementias and heart disease, although short-stay residents are more likely to have diabetes.^10^ Previous studies concerning nursing home resident hurricane exposure examined only long-stay residents.^2,3,6,7^

Hurricane Irma, a large storm that struck the west coast of Florida in 2017, provides us with an opportunity to assess the role of patient characteristics not examined in previous research concerning the outcomes of exposed nursing home residents. Hurricane Irma made landfall in Cudjoe Key, Florida on September 10, 2017, as a Category 4 storm with winds in excess of 130 mph.^11^ Its immense size, measuring 400 miles across before landfall, ensured that the entire peninsula of Florida was affected.^11^ It was reported to have caused at least 1 direct and 33 indirect deaths in South Florida, including the subsequent heat-exposure deaths of 14 residents at the Rehabilitation Center at Hollywood Hills.^11^ Given previous research suggesting that the deaths occurring in the weeks and months after a hurricane exceed official reports,^3^ this study seeks to use Medicare claims data and nursing home administrative data to estimate the 30- and 90-day mortality and morbidity associated with Hurricane Irma among nursing home residents. Specific attention was paid to the association of Irma with morbidity and mortality among short-stay vs long-stay residents in the nursing home cohort in Florida. This study aims to identify the degree to which hurricane disasters are associated with outcomes among nursing home residents beyond what is initially reported; findings may help to improve preparedness efforts to protect those who may be most at risk of harm.

## Methods

### Data Sources

We used the Centers for Medicare and Medicaid Services Standard Analytical Files combined with the Minimum Data Set to create a cohort of data on nursing home residents residing in Florida facilities during calendar years 2015 and 2017. The Standard Analytical Files are part of the Limited Data Set and contain records on Medicare claims such as hospitalization, hospice status, and mortality. The Minimum Data Set 3.0 is a federally mandated assessment of nursing home residents’ personal health history with information on comorbidities, functional level, and cognitive status.^12^ Data are collected quarterly on all nursing home residents and are also collected after significant changes in health care status. The institutional review boards at Brown University and the University of South Florida approved this study and provided a waiver of informed consent for secondary data under 45 CFR 46.116.

### Study Population

We identified a study population of nursing home residents exposed to Hurricane Irma.^6^ Hurricane Irma made landfall in Florida on September 10, 2017, but its vast size affected the entirety of the state of Florida, and its predicted point of landfall remained unclear until just before the storm. Our exposure cohort was therefore defined as all Florida residents of nursing homes as of September 7, 2017. We excluded residents younger than age 65 years (n = 6341) as their health care outcomes are not captured with Medicare claims. We chose this date as 3 days before landfall to capture health care outcomes related to evacuation. Previous research suggests that nursing home administrators generally make decisions around 72 hours before the storm on whether to evacuate or shelter.^13^ Finally, we characterized residents as short-stay vs long-stay based on their duration of residence at the nursing home. Long-stay residents were defined as having resided at the facility for at least 90 days before to September 1. We used these same criteria when creating the comparison cohort. The comparison cohort contained all nursing home residents residing in Florida nursing homes as of September 7, 2015, excluding residents younger than age 65 years (n = 7196). We choose the year 2015 as a comparison year due to lack of similar activity in 2015 compared with Hurricane Matthew and Hurricane Hermine in 2016.

### Outcomes

Our dependent variables included an assessment of mortality and first hospitalization. We used 30- and 90-day windows for identifying outcomes based on previous research that showed diminishing effects of storms after 180 days.^3^

### Statistical Analysis

We calculated descriptive statistics on study population demographic characteristics using the Minimum Data Set. We determined proportions and means for each cohort by long-stay and short-stay status of the resident and calculated respective *P* values using general estimating equations to cluster for persons who were in years 2015 and 2017 and by nursing home facility. General estimating equations uses a sandwich estimator that was specifically designed to handle and adjust for correlations within nested data. To reduce the strong bias toward type I error in this study, whereby *P* values are spuriously low due to extremely large population size, we used the 2-tailed *P* value of .01 (rather than the conventional .05) as the threshold for statistical significance.

We used the Standard Analytical Files to determine the 30- and 90-day total health outcomes of each cohort after the September 7 date for each respective year. We calculated the first hospitalization cumulative incidence and the mortality rate by dividing the total outcomes at 30- and 90-day by the total population on September 7 of the respective year. We adjusted the cumulative incidence and mortality rates for nursing home facility and calculated 95% CIs, displaying the final numbers per 1000 nursing home residents. We determined excess morbidity and mortality by calculating the risk difference for both first hospitalization and mortality between 2015 and 2017 and multiplying it by the total population in 2017. We also used general estimation equations to estimate the odds of mortality or first hospitalization in 2017 compared with 2015. We clustered the model by person ID, to account for persons who were in 2015 and 2017, and by facility ID. We have included a directed acyclic graph (DAG) as eFigure in the Supplement to show our analysis assumptions. All analyses were performed in SAS version 9.0 (SAS Institute Inc). Data were analyzed from August 28, 2019, to July 22, 2020.

## Results

A total of 67 835 residents of nursing homes were identified in the exposure cohort in 2017. We excluded 6271 residents (9.2%) who were younger than 65. Our final exposure cohort included 61 564 nursing home residents who were present in 640 Florida nursing home facilities on September 7, 2017. For our comparison sample, we identified 68 921 residents as of September 1, 2015. We excluded 7108 (11.5%) residents based on similar exclusions for a final comparison cohort of 61 813 residents. Table 1 contains baseline demographic characteristics and health characteristics of all nursing home residents in Florida in 2015 (comparison) and 2017 (exposure) by stay status (long vs short). In general, there were more long-stay residents compared with short-stay, with 43 258 and 43 168 long-stay residents compared with 18 555 and 18 396 short-stay residents in 2015 and 2017, respectively. Most residents were female (2015, 68%; 2017, 67%), mostly White (2015, 79%; 2017, 78%), and approximately 40% of the residents in each group were over the age of 85 years. We found that Changes in Health, End-stage Disease, and Signs and Symptoms scores^14^ were higher among long stay residents (for example, mean [SD] for short stay 2015 was 0.65 [0.87] and for short stay 2017, 0.65 [0.86]; *P* > .99; mean [SD] for long stay 2015, 0.81 [0.96], and for long stay 2017, 0.76 [0.94]; *P* < .001) and that activity of daily living scores were comparable. Both the average number of days in the nursing home before the storm and the number of acute first hospitalization in the month before the storm differed among short stay residents in 2015 and 2017 but not among long-stay residents.

**Table 1.** Baseline Demographic and Health Characteristics of All Nursing Home Residents in Florida in 2015 and 2017

Stay type	Short stay^a^			Long stay^d^		
	2015 (N = 18 555)	2017 (N = 18 396)	*P* value^b^^,^^c^	2015 (N = 43 258)	2017 (N = 43 168)	*P* value^b^^,^^c^
Sex, No. (%)			.09			.02
Female	11 499 (61.97)	11 243 (61.12)		30 254 (69.94)	29 925 (69.32)	
Male	7056 (38.03)	7153 (38.88)		13 004 (30.06)	13 243 (30.68)	
Age, No. (%)^e^			.04			<.001
65-74 y	4529 (24.41)	4685 (25.47)		7930 (18.33)	8597 (19.92)	
75-84 y	6622 (35.69)	6501 (35.34)		13 575 (31.38)	13 637 (31.59)	
≥85 y	7404 (39.90)	7210 (39.19)		21 753 (50.29)	20 934 (48.49)	
Race, No. (%)			.67			<.001
White	15 466 (83.35)	15 250 (82.90)		33 540 (77.53)	33 119 (76.72)	
Black	1972 (10.63)	2078 (11.30)		6762 (15.63)	6900 (15.98)	
Hispanic	828 (4.46)	708 (3.85)		2193 (5.07)	2277 (5.27)	
Other	289 (1.56)	360 (1.95)		763 (1.77)	872 (2.03)	
Comorbidity						
CHESS, mean (SD)	0.65 (0.87)	0.65 (0.86)	>.99	0.81 (0.96)	0.76 (0.94)	<.001
Missing, No. (%)	5907 (31.83)	7210 (39.19)		2287 (5.28)	3997 (9.26)	
ADL, mean (SD)	17.84 (4.48)	17.67 (4.44)	<.001	17.99 (6.33)	17.83 (6.15)	<.001
Missing, No. (%)	3726 (20.08)	5777 (31.40)		1577 (3.65)	3195 (7.40)	
Days in nursing home before storm, mean (SD)	30 (25)	28 (24)	<.001	1031 (978)	1034 (1004)	.53
Acute first hospitalizations in month before storm, No. (%)	11 407 (61.48)	11 769 (63.98)	<.001	2339 (5.41)	2320 (5.37)	.71

Abbreviation: ADL, activity of daily living; CHESS, Changes in Health, End-stage Disease, and Signs and Symptoms (range, 0-5, with higher scores indicating worse prognosis).

^a^ Short stay indicates less than 90 days.

^b^ *P* values were derived using general estimating equations to account for persons in Florida nursing home in 2015 and 2017 and clustering by nursing home.

^c^ Given the large size of the population, and the strong bias toward a type I error, a 2-tailed *P* = .01 level was considered as the threshold for statistical significance.

^d^ Long stay indicates 90 days or more.

^e^ Excluding those younger than age 65 years.

### Hospitalizations and Mortality Data

Table 2 compares the number of first hospitalizations and deaths in the exposure and comparison cohorts at 30 and 90 days following the storm. We found the total number of first hospitalizations and total number of deaths increased in 2017 compared with 2015. Table 3 displays the cumulative incidence, mortality rate, and odds ratios (ORs) of first hospitalization and mortality in residents exposed to Hurricane Irma compared with the control cohort. The odds of a first hospitalization for those exposed (vs nonexposed) were 1.09 (95% CI, 1.05-1.13) within the first 30 days poststorm and 1.05 (95% CI, 1.02-1.08) at 90 days; the odds of mortality were 1.12 (95% CI, 1.05-1.18) at 30 days and 1.07 (95% CI, 1.03-1.11) at 90 days. We calculated the risk difference to determine that in 2017, there were an additional 437 and 554 hospitalizations in the 30-day and 90-day poststorm period, respectively, compared with the nonstorm 2015 comparison cohort. We also found in 2017 there were an additional 262 and 433 deaths in the 30-day and 90-day poststorm period, respectively, compared with 2015 (Figure).

**Table 2.** Number of First Hospitalizations and Mortality

Variable	No. (%)	
	2015 (N = 61 813)	2017 (N = 61 564)
**First hospitalizations**		
Within 30 d		
All	5029 (8.14)	5446 (8.85)
Short stay^a^	2942 (4.76)	3144 (5.11)
Long stay^b^	2087(3.38)	2302 (3.74)
Within 90 d		
All	10 954 (17.72)	11 462 (18.62)
Short stay^a^	5708 (9.23)	5878 (9.54)
Long stay^b^	5246 (8.49)	5584 (9.08)
**Mortality**		
Within 30 d		
All	2268 (3.67)	2521 (4.09)
Short stay^a^	1131(1.83)	1183 (1.92)
Long stay^b^	1137(1.84)	1338 (2.17)
Within 90 d		
All	6154 (9.96)	6563 (10.66)
Short stay^a^	2742 (4.44)	2841 (4.61)
Long stay^b^	3412(5.52)	3722 (6.05)

^a^ Short stay indicates less than 90 days.

^b^ Long stay indicates 90 days or more.

**Table 3.** First Hospitalization Incidence Rate and Mortality Rate^a^

Variable	Rate (95% CI)		Odds ratio (95% CI)^b^
	2015	2017	
First hospitalization			
Within 30 d	81.36 (79.23-83.54)	88.46 (86.25-90.73)	1.09 (1.05-1.13)
Within 90 d	177.2 (174.2-180.2)	186.2 (183.1-189.3)	1.05 (1.03-1.08)
Mortality			
Within 30 d	36.69 (35.24-35.24)	40.95 (39.41-42.54)	1.12 (1.06-1.18)
Within 90 d	99.56 (97.23-101.9)	106.6 (104.2-109.1)	1.07 (1.04-1.11)

^a^ Rates are calculated per 1000 nursing home residents and are clustered by person ID and nursing home facility ID.

^b^ The odds ratio represents the odds of mortality or hospitalization for a nursing home resident in 2017 compared with 2015, clustered for person ID and nursing home facility ID.

**Figure.**
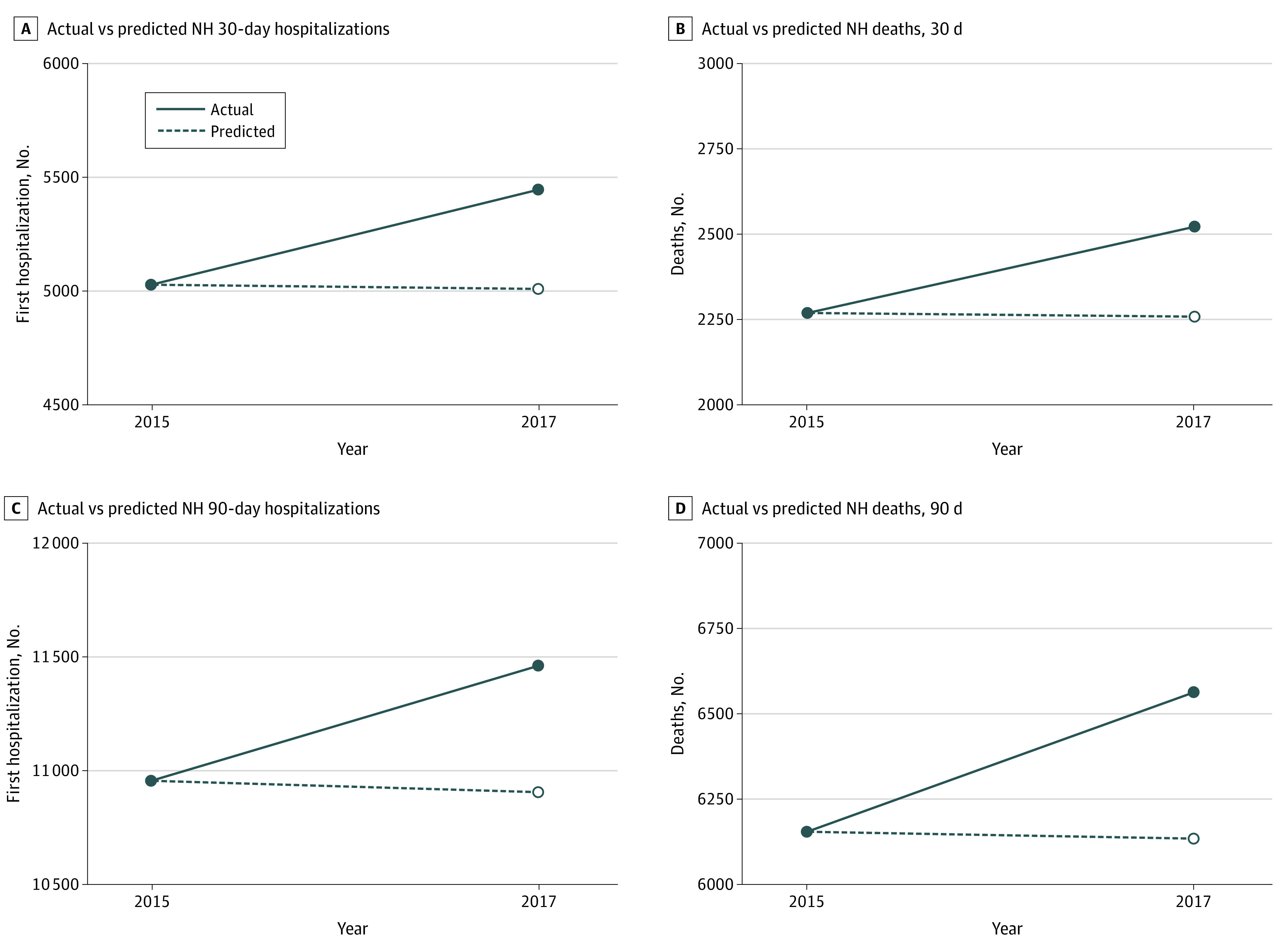
Additional First Hospitalization and Mortality in 2017 at 30-Day and 90-Day Intervals Compared With 2015^a^

### Short- vs Long-Stay Residents

Long-stay residents exposed to Hurricane Irma were at particular risk for first hospitalization and death (Table 4). Among long-stay residents, the odds of mortality for those exposed to Hurricane Irma were 1.18 (95% CI, 1.08-1.29) times those unexposed and the odds of hospitalization were 1.11 (95% CI, 1.04-1.18) times those unexposed in the post 30-day period. In contrast, exposure to Hurricane Irma was not associated with odds of mortality among short-stay residents at 30 (OR, 1.06; 95% CI, 0.98-1.14) or 90 days (OR, 1.05; 95% CI, 1.00-1.10) compared with the comparison cohort. Those short-stay residents who were exposed, however, were significantly more likely to be hospitalized by 8% (OR, 1.08; 95% CI, 1.03-1.13) at 30 days and 4% (OR, 1.04, 95% CI, 1.01-1.07) at 90 days. We conducted a sensitivity analysis by running models controlled for possible confounders that we selected from variables in Table 1 that we determined to be statistically different (*P* < .01) between 2015 and 2017. While our 95% CIs slightly widened, our point estimates stayed similar to the unadjusted model (eTable in the Supplement). One difference was that after adjusting for age group and activity of daily living score, we found that the odds of first hospitalization at 90 days for long-stay residents exposed to Hurricane Irma were 1.03 (95% CI, 0.99-1.06) times those unexposed. However, this may be due to missing data in the activity of daily living score.

**Table 4.** First Hospitalization Incidence Rate and Mortality Rate at 30- and 90-Day Intervals and Odds Ratios Among Long-Stay and Short-Stay Residents

Variable	Rate (95% CI)^a^		Odds ratio (95% CI)^b^
	2015	2017	
**First hospitalization**			
Within 30 d			
Short stay^c^	158.6 (153.4-163.9)	170.9 (164.7-177.3)	1.08 (1.03-1.13)
Long stay^d^	48.25 (46.27-50.31)	53.33 (51.25-55.49)	1.11 (1.04-1.17)
Within 90 d			
Short stay^c^	307.6 (301.1-314.3)	319.5 (312.9-326.3)	1.04 (1.01-1.07)
Long stay^d^	121.3 (118.2-124.4)	129.4 (126.2-132.6)	1.07 (1.03-1.10)
**Mortality**			
Within 30 d			
Short stay^c^	60.95 (57.61-64.50)	64.31 (60.86-67.95)	1.06 (0.98-1.14)
Long stay^d^	26.28 (24.82-27.84)	31.00 (29.40-32.67)	1.18 (1.09-1.28)
Within 90 d			
Short stay^c^	147.8 (142.8-153.0)	154.4 (149.3-159.7)	1.05 (1.00-1.10)
Long stay^d^	78.88 (76.38-81.46)	86.22 (83.61-88.91)	1.09 (1.05-1.14)

^a^ Rates are calculated per 1000 nursing home residents and are clustered by person ID and nursing home facility ID.

^b^ The odds ratio represents the odds of mortality or hospitalization for a nursing home resident in 2017 compared with 2015, clustered for person ID and nursing home facility ID.

^c^ Short stay indicates less than 90 days.

^d^ Long stay indicates 90 days or more.

## Discussion

Hurricane Irma was associated with significant increases in mortality and hospitalization among the 61 564 nursing home residents in Florida nursing home. Compared with 2015, we identified an additional 262 nursing home deaths at 30 days post exposure and 433 more deaths at 90 days. The number of 30-day postexposure deaths of nursing home residents is noteworthy because it is 139 higher than the 123 deaths reported by the Centers for Disease Control and Prevention (CDC) for the entire population of Florida.^15^ The methods used by the CDC to examine vital statistics death data from the electronic death registration system evaluates direct and indirect deaths attributed to the hurricane. The CDC total does include deaths resulting from the exacerbation of existing medical conditions. However, we believe its conclusions undervalue indirect deaths in the long-term care setting in which attribution of death to natural causes might be more likely to occur, even for those who died from worsening comorbidities.

This finding is consistent with previous research involving Hurricanes Katrina, Rita, Gustav, and Ike, which showed that the actual effects of mortality and morbidity post storm on the nursing home cohort was also significantly higher than originally recorded.^3^ Collectively, these results underscore the susceptibility of nursing home residents to destabilizing disasters such as hurricanes and the more recent COVID-19 crisis.^16^ These findings add context to the situation facing nursing home residents during the COVID-19 crisis. Nursing home residents have been shown to be significantly more at risk for mortality from COVID-19.^16^ Although hurricane disasters are geographically more localized than the COVID-19 pandemic, there are similarities in their disruption of usual nursing home care.

This study found that long-stay residents experienced disproportionately greater morbidity and mortality associated with the hurricane. Long-stay residents often have significant cognitive and functional impairments, making the care disruptions that are inevitable during storms more taxing to residents and staff.^13,17^ Long-stay residents are also likely to be more at risk for the transfer trauma associated with evacuation and to poststorm heat exposure such as the conditions that occurred at the Rehabilitation Center at Hollywood Hills in Broward County post storm.^18,19^ It is noteworthy that Florida has recently instituted a emergency power rule to ensure that nursing homes have the capacity to provide an environment where temperatures do not go above 81 °F for at least 96 hours in the event of a power outage.^20^ Previous research on heat exposure and frail populations, including those exposed to historical heat waves in Chicago in 1995 and in Europe during 2003, show significant increases in mortality among those who are bedbound or frail.^21,22,23,24^ Future research exploring whether excess mortality can be attributed to specific patient characteristics, to the evacuation process itself, to delays in receiving appropriate health care services, or to post disaster consequences such as heat exposure among residents of facilities that lost power in the days following the storm is warranted.

An additional factor that may contribute to excess hospitalization or mortality during a hurricane is the availability and adequacy of direct-care nurse staffing. Qualitative studies suggest that obtaining adequate direct-care nurse staffing may be difficult during hurricanes due to shortages and absenteeism,^25,26^ notable because of the association between nurse staffing levels and resident quality of care.^27,28^ Although the staffing-quality relationship is dependent on the staff type (eg, registered nurse vs certified nursing assistant), the outcome of interest (eg, physical restraints vs pressure ulcers), or the methodologic rigor of the study (ie, controlling for endogeneity and other forms of bias),^29^ direct-care staffing may be of greater importance specifically during hurricanes when frail residents may experience the storms’ physical and psychological force. Further study regarding the role of staffing in morbidity and mortality is warranted.

### Limitations

There are limitations to this work. First, our data were based on a comparison between 2 years of data, specifically 2017 (exposure) and 2015 (control). We selected 2015 as our comparison year for its lack of hurricanes. Research has reported geographic differences to annual mortality and morbidity rates.^30^ In the nursing home setting, differential flu rates can account for changes in morbidity and mortality.^31^ According to the Florida Department of Health’s Florida Flu Review, however, rates of flu remained low during September of 2015 and 2017 with slightly more activity in 2015 than our exposure year. Nevertheless, these differences might be associated with other factors despite the storm.^32^ Finally, it is likely that not all of the mortality and morbidity increases were directly attributable to exposure to the storm. Certainly, hurricane disasters are significant for their considerable disruption to the public health infrastructure. Crowded hospitals, reduced access to care, and reductions in staffing at the nursing home due to mandatory public evacuations are likely to be key components of the differential morbidity, but those topics are beyond the scope of this study.

## Conclusions

This study provides the health outcomes of nursing home residents who experienced Hurricane Irma. There were considerable increases in mortality among the nursing home population. Moreover, although nursing home residents represent only a small portion of Florida’s population, our analysis shows substantially higher counts of death than were reported by the CDC for the entire state. Our finding that long-stay residents experienced greater mortality and morbidity associated with the storm is important for public health officials who must prioritize this frail population for heightened emergency preparedness in disaster situations.

## Notes

Supplement. **eFigure.** Directed Acyclic Graph (DAG) to Demonstrate Causal Assumptions in the Analysis **eTable.** Adjusted First Hospitalization Incidence Rate and Mortality Rate at 30- and 90-day Intervals and Odds Ratios Among Long-Stay (LS) and Short-Stay (SS) Residents
