# Supplementary material for: Association Between Exposure to Hurricane Irma and Mortality and Hospitalization in Florida Nursing Home Residents
Source: JAMA Netw Open. 2020 Oct 6;3(10):e2019460. doi: 10.1001/jamanetworkopen.2020.19460 (PMC7539118; doi:10.1001/jamanetworkopen.2020.19460)
Supplement: Supplement. — eFigure. Directed Acyclic Graph (DAG) to Demonstrate Causal Assumptions in the Analysis eTable. Adjusted First Hospitalization Incidence Rate and Mortality Rate at 30- and 90-day Intervals and Odds Ratios Among Long-Stay (LS) and Short-Stay (SS) Residents [file jamanetwopen-e2019460-s001.pdf]

.

Supplemental Online Content

Dosa DM, Skarha J, Peterson LJ, et al. Association between exposure to Hurricane Irma and mortality and hospitalization in Florida nursing home residents. *JAMA Netw Open*. 2020;3(10):e2019460. doi:10.1001/jamanetworkopen.2020.19460

eFigure. Directed Acyclic Graph (DAG) to Demonstrate Causal Assumptions in the Analysis

eTable. Adjusted First Hospitalization Incidence Rate and Mortality Rate at 30- and 90-day Intervals and Odds Ratios Among Long-Stay (LS) and Short-Stay (SS) Residents

This supplemental material has been provided by the authors to give readers additional information about their work.

© 2020 Dosa DM et al. *JAMA Network Open*.

.

eFigure. Directed acyclic graph (DAG) to demonstrate causal assumptions in the analysis[a]

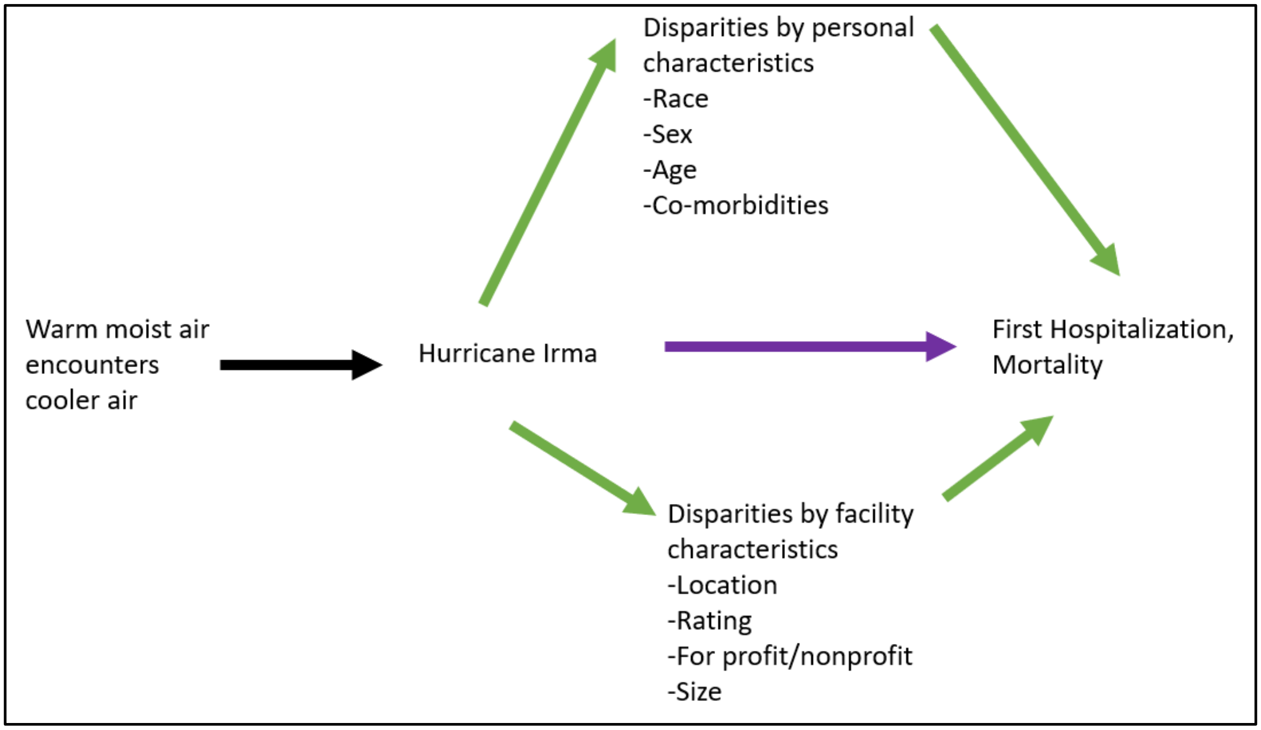

[a]To expand our discussion on causality we have created a Directed Acyclic Graph (DAG) (Supplemental Figure 1) to show our assumptions and justify our analysis. These are commonly used in epidemiological studies to determine what variables are confounders. We determine confounders by tracing pathways by following arrows. Arrows in DAGs represent direct causal effects of one factor on another. If arrows follow each other then they are on the causal pathway and are not confounders (Ex: Poverty -> Smoking -> Lung Cancer). If arrows don't follow each other, they may be a confounder (Coffee Drinking <- Smoking -> Lung Cancer). In terms of our study, it is good to think of it in terms of what causes a hurricane (ie. warm air mixes with cold air). When we write out our arrows we will never write Disparities by Race -> Hurricanes Exposure. Rather we would write Hurricane Exposure -> Disparities by Race. Our goal for this analysis was to capture the Total Effects of Hurricane Irma on mortality and first hospitalization in nursing homes in Florida. The DAG demonstrates the Direct effects (green pathway) and Indirect effects (purple pathways) that sum (Indirect + Direct) to the Total Effect. Since personal and facility characteristics are on the causal pathway they are not confounders and thus should not be controlled for in our analysis.

© 2020 Dosa DM et al. *JAMA Network Open*.

.

eTable. Adjusted first hospitalization incidence rate and mortality rate at 30 and 90 day intervals and odds ratios among Long-Stay (LS) and Short-Stay (SS) residents[a,b,c]

	2015 Rate, 95% CI	2017 Rate, 95% CI	Odds Ratio, 95% CI
	First Hospitalization		
Within 30 days			
SS	158.6 (153.4, 163.9)	170.9 (164.7, 177.3)	1.07 (1.01, 1.13)
LS	48.25 (46.27, 50.31)	53.33 (51.25, 55.49)	1.07 (1.00, 1.13)
Within 90 days			
SS	307.6 (301.1, 314.3)	319.5 (312.9, 326.3)	1.04 (1.00, 1.08)
LS	121.3 (118.2, 124.4)	129.4 (126.2, 132.6)	1.03 (0.99, 1.06)
	Mortality		
Within 30 days			
SS	60.95 (57.61, 64.50)	64.31 (60.86, 67.95)	1.04 (0.95, 1.15)
LS	26.28 (24.82, 27.84)	31.00 (29.40, 32.67)	1.21 (1.11, 1.31)
Within 90 days			
SS	147.8 (142.8, 153.0)	154.4 (149.3, 159.7)	1.03 (0.97, 1.09)
LS	78.88 (76.38, 81.46)	86.22 (83.61, 88.91)	1.12 (1.07, 1.17)

[a]Rates are calculated per 1,000 nursing home residents and are clustered by person ID and nursing home facility ID

[b]The odds ratio represents the odds of mortality or hospitalization for a nursing home resident in 2017 compared to 2015, clustered for person ID and nursing home facility ID, and adjusted for variables that showed statistically significant differences (<.01) in Table 1. For short-stay residents, models were controlled for ADL score and average number of days in NH prior to storm. For long-stay residents, models were controlled for age group and ADL score

[c]Long-stay residents $\geq$ 90 days while short-stay residents are < 90 days

© 2020 Dosa DM et al. *JAMA Network Open*.
